# Cardiac Sarcoidosis: Is it More Common in Men?

**DOI:** 10.1007/s00408-015-9805-8

**Published:** 2015-09-28

**Authors:** Magdalena M. Martusewicz-Boros, Piotr W. Boros, Elżbieta Wiatr, Anna Kempisty, Dorota Piotrowska-Kownacka, Kazimierz Roszkowski-Śliż

**Affiliations:** 3rd Lung Diseases Department, National Research Institute of TB & Lung Diseases, Plocka 26, 01-138 Warsaw, Poland; Lung Pathophysiology Department, National Research Institute of TB & Lung Diseases, Plocka 26, 01-138 Warsaw, Poland; 1st Lung Diseases Department, National Research Institute of TB & Lung Diseases, Plocka 26, 01-138 Warsaw, Poland; 1st Department of Clinical Radiology, Medical University of Warsaw, Chalubinskiego 5, 02-004 Warsaw, Poland

**Keywords:** Sarcoidosis, Heart involvement in systemic diseases, Cardiac sarcoidosis, Sex distribution, Epidemiology

## Abstract

**Introduction:**

Sarcoidosis is a systemic granulomatous disease which predominantly affects the lungs, although granulomas can also involve all other organs, including the heart. Cardiac sarcoidosis (CS) may occur at any stage of the disease and may be the cause of sudden cardiac death, even in a previously asymptomatic patient. The aim of this study was to evaluate the incidence of CS in a large group of patients diagnosed or followed up due to sarcoidosis.

**Methods:**

We performed a retrospective analysis of patients at our institution discharged with the final diagnosis “sarcoidosis” (ICD-10: D86) from January 2008 to October 2012. Only those with biopsy (from respiratory tract or lymph nodes) confirmed diagnosis of sarcoidosis were included. We then selected the subset of patients with cardiac involvement due to sarcoidosis confirmed by positive magnetic resonance imaging.

**Results:**

The study covered 1375 consecutive sarcoidosis patients (51 % men), who were hospitalized during 5 years. Multiorgan disease was detected in 160 cases (11.7 %), and cardiac involvement was found in 64 patients (4.7 % of all), 70.3 % of whom were men. Twelve of those with CS were in stage I, 48 in stage II, and four in stage III. The odds ratio for having cardiac involvement in men compared to women was 2.3 (95 % CI 1.36–4.0, *p* = 0.002).

**Conclusions:**

Cardiac involvement in sarcoidosis was diagnosed in the similar percentage as in previously published data but was significantly more frequently in men.

## Introduction

Sarcoidosis is a systemic granulomatous disease with predominant manifestation in the chest, often presenting as bilateral hilar lymphadenopathy with or without pulmonary infiltrates. Despite numerous hypotheses, the etiology of sarcoidosis still remains unclear. An unspecified antigen in individuals genetically predisposed induces an abnormal immune response. The consequence is an inflammatory process with formation of sarcoidosis granulomas. While more than 90 % of patients with sarcoidosis have lung involvement, granulomas can also involve any other organs, including the heart [1].

Cardiac sarcoidosis (CS) is usually ascertained in about 5 % of patients with already diagnosed sarcoidosis of the respiratory system but may be detected without pulmonary disease [2, 3]. An autopsy study reported cardiac involvement in 27 % of cases in United States or Europe [3–6]. These differences demonstrate the diagnostic difficulties, which are still present. Heart involvement is the primary cause of poor outcome in sarcoidosis, as may be the cause of sudden cardiac death, even in previously asymptomatic patients. CS may also cause heart failure. Early diagnosis of the CS and the early initiation of corticosteroid therapy seem to improve the prognosis [7–9]; however, extent involvement may correlate with the absence of functional improvement and high incidence of adverse outcomes [10]. Age gender and race have all been identified as factors associated with specific clinical presentations of sarcoidosis; however, demographic features that predict cardiac involvement remain elusive [3].

The National Tuberculosis & Lung Diseases Research Institute in Warsaw, Poland serves as the regional referral center for patients with sarcoidosis and other interstitial lung diseases. This provided the opportunity to describe the incidence of clinically important cardiac involvement in a much larger group of patients with sarcoidosis than previously described [11–15].

The main purpose of this investigation was to evaluate the incidence of CS in a large group of patients diagnosed or followed up due to sarcoidosis and looking for specific pattern of patients diagnosed with heart involvement.

## Materials and Methods

We performed retrospective analysis of database discharged patients with the final diagnosis “Sarcoidosis of other and combined sites” (ICD-10: D86.8). Only patients with biopsy (from lungs or lymph nodes) confirmed diagnosis of sarcoidosis were included. We than selected the subset of patients with cardiac involvement due to sarcoidosis, who were identified when partially or fully complied with criteria presented in Modified Guidelines for Diagnosis of Cardiac Sarcoidosis based on the Study Report on Diffuse Pulmonary Diseases from the Japan Ministry of Health and Welfare, 1993 (9) and modifications JMHW 2006 (Table 1). However, the diagnosis was confirmed finally by a positive magnetic resonance imaging (MRI) test.

**Table 1:** Revised guidelines for diagnosing CS 2006 (Japan Society of Sarcoidosis and Other Granulomatous Disorders)

1. Histologic diagnosis group
Cardiac sarcoidosis is confirmed when myocardial biopsy specimens demonstrate noncaseating epithelioid cell granulomas with histological or clinical diagnosis of extracardiac sarcoidosis.
2. Clinical diagnosis group
Cardiac sarcoidosis is diagnosed in the absence of a cardiac biopsy when extracardiac sarcoidosis is diagnosed histologically or clinically and satisfies the following conditions and more than one in six basic diagnostic criteria.
(1) More than two of four major criteria are satisfied, or
(2) One in four major criteria and more than two in five minor criteria are satisfied.
*Major criteria*
(a) Advanced AV block
(b) Basal thinning of the interventricular septum
(c) Positive cardiac gallium uptake
(d) Left ventricle ejection fraction less than 50 %
*Minor criteria*
(a) Abnormal ECG findings: Ventricular arrhythmias (VT, multifocal or frequent PVCs), CRBBB, axis deviation or abnormal Q-wave
(b) Abnormal echocardiography: Regional abnormal wall motion or morphological abnormality (ventricular aneurysm, wall thickening)
(c) Perfusion defect detected by 201Tl myocardial scintigraphy or 99Tc myocardial scintigraphy
(d) Gd-enhanced MRI: Delayed enhancement of myocardium.
(e) Endomyocardial biopsy: Interstitial fibrosis or monocyte infiltration over moderate grade

*AV* atrioventricular, *ECG* electrocardiogram, *VT* ventricular tachycardia, *PVCs* premature ventricular contractions, *MRI* magnetic resonance imaging, *CRBBB* complete right bundle branch block

The analysis covered the period from January 2008 to October 2012 in 1st and 3rd Lung Diseases Departments of the National Tuberculosis & Lung Diseases Research Institute in Warsaw. We collected demographic information, medical history, including comorbidities, and medical treatment. Patients with a history of coronary artery disease (CAD) or other heart disease which could influence the MRI examination making it non-conclusive were not included. However, in a few cases, when MRI investigation was suggestive for CAD, but without previous history, coronary angiography was performed (in all cases it was negative and patients were included into study). The evaluation of all patients included laboratory tests (e.g., among others determination of the highest serum levels of angiotensin-converting enzyme, calcium metabolism), radiological examination, electrocardiography (ECG) and often echocardiography (ECHO), pulmonary function tests, and an ophthalmology assessment (or other consultation of specialist, if needed).

Cardiac evaluations were usually initiated because of tachycardia, confusion, syncope, chest discomfort, or fatigue; or abnormalities seen on an ECG or ECHO. In cases with suspected CS, 24 h-Holter monitoring and delayed enhancement cardiovascular MRI were performed using a 1.5 T scanner (GE Signa Excite 11) using a torso phased-array coil. Each MRI included cine imaging with steady-state free precession sequence (SSFP; FIESTA), black blood imaging with FSE-XL breath-hold sequences (double and triple inversion recovery (IR) pre-pulse), and delayed enhancement imaging using T1-weighted, IR gradient echo sequence 15–20 min after intravenous administration of 0.1 mmol/kg Gd-based contrast agent. Images were obtained in standard long axis, short axis, and four-chamber views using ECG gating. All tests were performed using the same equipment/procedure, and results were described by the same well experienced specialist.

The nature of this study is a non-interventional, descriptive, retrospective analysis of de‐identified data, obviating the need for approval from local Ethics Committee.

### Statistical Analysis

Descriptive data were presented as mean ± SD and range or 90 % CI where indicated. Group comparisons were made using paired t-tests for independent samples. The incidence ratios were presented as numbers of patients in groups and percentages. The *χ*^2^ test was used to test for differences in the prevalence of observations. Statistical analyses were performed using STATISTICA (data analysis software system StatSoft, Inc. 2010), version 9.1.

## Results

**Fig. 1:**
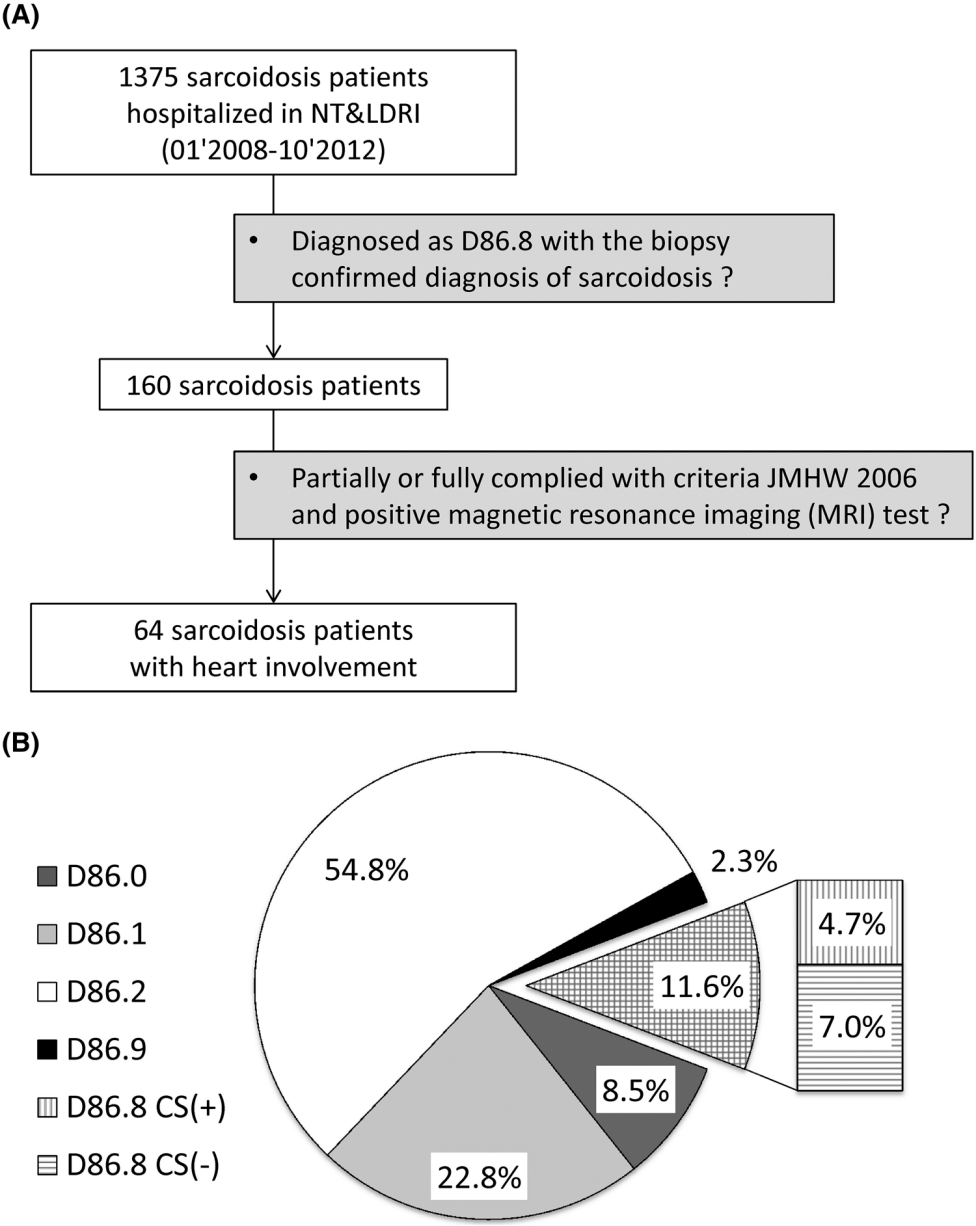
**a** The flowchart of the study. **b** The distribution of diagnosis of sarcoidosis according to ICD-10 classification, where D86.0 -Sarcoidosis of lung; D86.1—Sarcoidosis of lymph nodes; D86.2—Sarcoidosis of lung with sarcoidosis of lymph nodes; D86.8—Sarcoidosis of other and combined sites; D86.9—Sarcoidosis unspecified; CS(+)—patients with diagnosis of cardiac sarcoidosis; CS(−)—patients without diagnosis of CS, data presented as numbers of cases and percentages

During the 5-year period, 1375 consecutive sarcoidosis patients were observed in two lung disease departments. The distribution of gender in the whole group was comparable, there were 671 female (49 %) and 704 males (51 %), in mean age: 43.7 ± 12.2 years (F: 48.0 ± 12.8; M: 39.6 ± 10.1). Multiorgan disease was detected in 160 cases (11.7 %). Cardiac involvement confirmed by MRI was found in 64 patients (4.7 % of the entire group). See Fig. 1 part A for flowchart of the study and part B for distribution of patients.

The most frequent sites, other than lung or heart involvement, were liver (18.8 %), spleen (18.8 %), and peripheral lymph nodes (17.2 %). The median time from the first diagnosis of sarcoidosis to detection of heart involvement was 18.8 months (range 0–9 years). All of the CS patients had affected lungs and/or intrathoracic lymph nodes. Twelve patients were in stage I, 48 in stage II, and four in stage III.

In 19 (29.7 %) cases, CS was diagnosed with radiological progression in the lungs, in 11 cases (17.2 %) during regression and in 34(53.1 %) at stabilization. 16 (25 %) patients had been previously treated with corticosteroids. 50 % of CS group (21 males and 11 females) were diagnosed because of symptoms (Table 2), while the others were identified due to ECG or ECHO abnormalities.

**Table 2:** Symptoms reported by patients with recognized CS

Symptoms	Number of symptomatic CS males = 21/45 (46.6 %)	Number of symptomatic CS females = 11/19 (57.8 %)
Sudden cardiac arrest	2 (4.4 %)	0
Syncope	3 (6.6 %)	3 (15.8 %)
Palpitations	9 (20 %)	7 (36.8 %)
Non-specific symptoms	7 (15.5 %)	4 (21 %)
Dyspnoea	4 (8.8 %)	2 (10.5 %)
Non-specific chest complaints	4 (8.8 %)	0

**Fig. 2:**
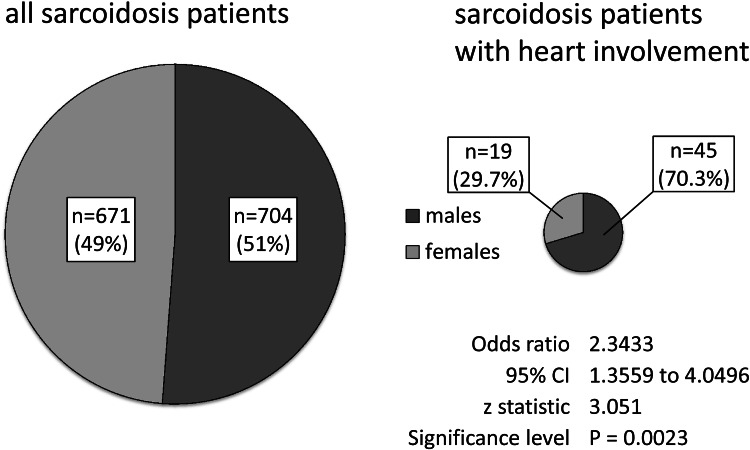
Sex distribution in all patients and in CS group (numbers and percentages)

The mean age of those with CS was 47 years (range: 25–77). There were 45 males and 19 females (70.3/29.7 %) in CS group. Odds ratio for having cardiac involvement in males comparing to females was 2.3; 95 % CI 1.4–4.0, *p* = 0.002 (see Fig. 2).

## Discussion

The diagnosis of CS remains difficult. There are no specific symptoms or signs. Most often heart involvement is clinically “silent” in the early stage of the disease. The initial presentation can be malignant arrhythmias, progressive heart failure, or sudden death [16–19]. This location of changes significantly worsens the prognosis of patients with sarcoidosis [20]. CS accounts for as many as 13–25 % (in United States) to 58–85 % of deaths (reported in Japan) from sarcoidosis [6, 21]. In addition to the asymptomatic course of the disease, also widely available tests like ECG or ECHO could be normal in the early stage of CS. Some cases with advanced heart involvement diagnosed postmortem had normal ECGs before death [6]. ECG changes, if any present, are non-specific. Conduction aberrations (from incomplete and complete bundle branch block 12–61 % to atrioventricular block of any degree 26–62 %) and (ventricular) arrhythmias (2–42 %) seem to be common [8, 11, 14, 22]. Holter monitoring is useful in clinical suspicion of CS [12]. Changes in ECHO can be seen with disease progression in 14–56 % [22–25]. Endomyocardial biopsy, despite high specificity, slack sensitivity (about 36 %), probably because of patchy involvement [26]. That is why this invasive procedure should not be a part of routine evaluation for patients with suspected CS [27].

No recommendations for the diagnosis and treatment of CS have been widely accepted. The diagnostic criteria developed by the Japanese Ministry of Health and Welfare (JMHW) in the absence of endomyocardial biopsy propose to identify cardiac involvement (Table 1). The development of imaging techniques allows the identification of changes at an earlier, less advanced stage of the disease [7, 13, 22, 28, 29]. Delayed enhancement cardiac MRI is regarded as very helpful and promising, and begins to pretend to be the gold standard for the diagnosis CS [7, 30, 31]. Recently published data and expert opinion also recommend use of MRI and positron emission tomography-computed tomography (PET-CT) as useful tools for detecting CS [32–34].

The incidence of CS in our study was lower than previously reported in postmortem or contrast-enhanced MRI based studies [5, 30, 35–38]. However, it should be noted that our study did not screen asymptomatic patients for CS but analyzed data only from symptomatic patients. Our study is the first epidemiological evaluation suggesting that CS is more common in men than women. A Japanese study reported a predominance of CS in women [39]. Other studies have shown no difference in CS rates between men and women [14, 40, 41], so our results may not be generalizable to patients from other countries. However, in recently published study performed in Europe, results were quite similar (male/female ratio 74/26 %), but due to smaller size of the group and male/female disproportion, this finding could not be regarded as statistically significant [36].

### Limitations of the Study

This is a retrospective review, with its inherent limitations. Sarcoidosis patients included to the study were hospitalized (no out-patients) in a large tertiary specialist center, what rise concern about pre-selection and possible bias; however, in Polish realities of health care funding, almost all patients are diagnosed in hospitals due to poor financing of diagnostic procedures in out-patients clinics. We did not perform coronary angiography studies to exclude CAD as a cause for delayed myocardial enhancement in all patients; however, patients with known history of CAD were excluded from the study. As only one person evaluated the MRI study inter-observer, and intra-observer agreement was not assessed.

## Conclusions

Clinically evident cardiac involvement in sarcoidosis was diagnosed in the similar percentage as in previously published data but was significantly more frequently in men. Further investigations are needed for factors predisposing to the occurrence of cardiac involvement in sarcoidosis and gender should be considered.
